# DIGIPART - A pilot dataset exploring the digitalisation of political parties

**DOI:** 10.1016/j.dib.2025.112173

**Published:** 2025-10-24

**Authors:** Marco Meloni, Fabio G. Lupato, Felix von Nostitz, Giulia Sandri, Oscar Barberà, Adrià Mompó

**Affiliations:** a University of Southampton. University Road, SO17 1BJ Southampton, United Kingdom; b Universidad Complutense de Madrid, *Av*/ Complutense s/n - 28040 Madrid, Spain; c ESPOL-LAB Université Catholique de Lille, 60 boulevard Vauban - CS 40109-59016 Lille Cedex, France; d Universitat de València, *Av*/ Tarongers s/n - 46022 València, Spain; e Universitat Oberta de Catalunya, Rambla del Poblenou 154-156 - 08018 Barcelona, Spain

**Keywords:** Political parties, Digital politics, Information and communication technologies, Online participation platforms, Democratic Innovations, Party organisation

## Abstract

The aim of this paper is to introduce the key features of the DIGIPART – Digitalisation in Parties dataset. The DIGIPART dataset (v.1.1) provides information on the digitalisation of several key dimensions of 76 statewide and non-statewide political parties through five major European countries (France, Italy, Germany, United Kingdom, and Spain) right after the COVID-19 pandemic (2021–22). The dataset is strongly informed by previous theorisations of party digitalisation (Fitzpatrick 2021 [1], García Lupato and Meloni 2023 [2]), which divide party digitalisation into different pillars: elections, deliberation, participation, resources, communication, and organisation. The information gathered on both party features and dimensions of party digitalisation allows to explore potential variables influencing and being influenced by such phenomenon. The information was gathered and coded between 2021 and 2022 by exploring parties’ websites and manifestos, as well as press releases and secondary sources. The DIGIPART dataset offers the possibility to quantify different dimensions of party digitalisation, many of them directly related to democratic innovations. The dataset was conceived as a pilot to then scale up to other countries and regions allowing further comparative research.

Specifications Table

**Table:** 

Subject	Social Sciences / Political Science
Specific subject area	The political structure of society. Party politics.
Type of data	Table (.xlsx)
Data collection	Data was gathered tracking public information disclosed by the parties themselves in their websites or manifestos between July 2021 and May 2022. Any initiative regarding digital tools introduction was registered as evidence of digitalisation. Mentions to digitalisation strategies in manifestos were also noted as proof of concern by the parties. Additionally, we consulted journals and press repositories when information was missing in the parties’ documents.
Data source location	Country: Spain Institution: Universidad Complutense de Madrid
Data accessibility	The dataset and codebooks are available via Zenodo. Repository name: Zenodo Data identification number: 10.5281/zenodo.10997699 v.1.2: 10.5281/zenodo.13918421 v.1.1: 10.5281/zenodo.10997782 v.0: 10.5281/zenodo.10997700 Direct URL to data: https://zenodo.org/records/13918421
Related research article	Sandri, G., Lupato, F. G., Meloni, M., von Nostitz, F., & Barberà, O. (2024). Mapping the digitalisation of European political parties. Information, Communication & Society, 1–22. https://doi.org/10.1080/1369118X.2024.2343369 [1] Mompó, A., Meloni, M., Barberà, O., Lupato, F., Sandri, G., & von Nostitz, F. (2025). When do parties go digital? Examining the drivers of internal and external party digitalisation. Party Politics. https://doi.org/10.1177/13540,688,251,339,977 [2]

## 1 Value of the Data

• Provides a complete map of the digitalisation of 76 statewide and non-statewide parties in five EU countries (France, Italy, Germany, United Kingdom, and Spain) in the early 2020s, right after the CODIV-19 pandemic.
• Operationalises key dimensions (elections, deliberation, participation, resources, communication, and organisation) of the digitalisation of political parties, while providing specific indicators and measures to quantify them.
• In a research avenue dominated by case studies, the dataset allows for the development of larger comparative studies and testing theoretical hypotheses in a way that has not been possible before.
• Makes possible the elaboration of several party digitalisation indexes, hence allowing for more systematic cross-sectional comparisons.
• The dataset provides a theoretically driven comparison of variables that have recently been implemented (or still are in several countries) and are strongly related to more inclusive ways of decision-making and participation.
• The release of the codebook and the dataset pave the way for the future coding of either more waves of data, more variables, and more countries across Europe and beyond.
• Combined with other datasets - whether focused on the party structure or the position and strength of political parties in a party (and political) system – It might allow to comprehensively understand the main impacts that the digital transformation might produce within political parties, in party competition or in other elements of the political system.

## 2 Background

The digitalisation of political parties is an ongoing trend that started by the mid 1990s in Western countries. There is a substantial amount of literature in the intersection between party politics and political communication that have focused, since the late 20th century, on the different uses and organisational impacts related to the use of digital technologies [3,4]. That said, a comprehensive account of the different party faces and dimensions affected by the digital transformation was missing till the early 2020s. One of the main theorisations came from Fitzpatrick’s Five Pillar model [5] that provided a thorough justification of five party dimensions and several subdimensions that could be transformed by the intensive use of ITCs (see also Table 2). She also discussed the state of the art and several research gaps (e.g. digital finances) on each dimension, hence setting the path for a future research agenda on the topic. Fitzpatrick’s model has been lately discussed and adapted by the literature to include alternative conceptions of the digital democracy [6].

Many of the dimensions and subdimensions included in the Five Pillar model are connected to more inclusive ways of decision-making and political participation within political parties. Some of the specific practices identified in the typology also constitute political innovations connected to the use of digital technologies. As a result, the Five Pillar Model also presented one of the most systematic catalogues of party digital democratic and participatory practices existing by the end of the 2010s. This is why the Five Pillar model has been adopted, with slight modifications, as the main theoretical source to build the DIGIPART dataset. As a short summary, the elections dimension focuses on the introduction, challenges and outcomes (e.g. turnout) connected to digital voting in internal procedures [7,8]. The second dimension explores how ITCs and digital tools might help to transform deliberation within parties, and their impacts [9,10]. The third dimension involves assessing to what extent the digital transformation is changing internal consultations, the advent of the multi-speed membership, and the emergence of new forms of membership involvement in election campaigns [[11], [12], [13]]. The resources dimension focuses on how digital tools are transforming party funding [14]. The use of digital tools for external communication has been extensively researched by literature [15,16].

A more thorough understanding of such dimensions has allowed to start building systematic qualitative comparisons of how some political parties are being reshaped by the digital transformation [[17], [18], [19]]. Such phenomenon has also been partially completed due to new information included in large international datasets such as the Political Parties Database [20,21]. While still being a pilot study, the DIGIPART dataset contains the more in-depth analysis related to party digitalisation in Europe [1,2].

## 3 Data Description

The Digitalisation in Parties (DIGIPART) dataset (v1.1) contains information on party digitalisation features collected after the COVID-19 pandemic (2021–2022), from 76 parties, from five major European countries: Germany, Italy, France, Spain, and the United Kingdom. The dataset is stored in Excel format (.xlsx) as well as the codebook in Zenodo (Table 1). A previous version of the DIGIPART (V0) dataset with lower units of analysis (*n* = 62 parties) is also available in Zenodo.

**Table 1:** List of DIGIPART files stored in Zenodo.

File name	Format type	File content
DIGIPART-Dataset_v.0	.xlsx	A single Excel sheet containing raw data on party digitalisation in Europe (*n* = 62)
DIGIPART-Codebook_Version-2.4	.pdf	The codebook used for coding party digitalisation
DIGIPART-Dataset_v.1	.xlsx	Updated version of the single Excel sheet (v.0) containing raw data on party digitalisation in Europe (*n* = 76)

DIGIPART provides basic data for the identification of the units of analysis: COUNTRY_ID provides a code for each country according to Eurostats Country Codes; COUNTRY refers to the abbreviation of the country name, following also Eurostats’ Country Codes; PARTY_ID, provides a code for each party; PARTY ACRONYM states the acronym in the original language; PARTY NAME states the full name of the party in English; YEAR OF FOUNDATION, refers to the year the party was registered; IDEOLOGY provides the Left-Right position of the party according to the Chapel Hill Experts Survey (coded 1–3) [22]; ELECTION YEAR indicates the year of the last general election; VOTES provides the percentage of votes, MPs includes the share of MPs that a party has in the Lower Chamber of the national parliament. Both data on votes and MPs are provided by the Parlgov database (https://www.parlgov.org/data-info/) [23] or press sources for those parties not included in the former.

The DIGIPART dataset provides information on six main dimensions (pillars) of party functions and activities: elections (EL), deliberation (DEL), participation (PART), resources (SOURCE), communication (COM) (Table 2). Each dimension is integrated by several (23) indicators.

**Table 2:** Dimensions and main variables of party digitalisation in the DIGIPART dataset.

Electoral pillar (EL)	Deliberative pillar (DEL)	Participatory pillar (PART)	Resources pillar (SOURCE)	Communication pillar (COM)
Online voting (leaders)	Deliberative online platform	Online consultations	Online fees’ payment	Party website
Online voting (candidates)	Other digital deliberative initiatives	Online membership	Online crowdfunding/ donations	Social media, SNS, MIMS
Online voting (party bodies)	Party Congress Digitalisation	Digital activities for electoral campaigning	Official online store	Other forms of online communication

Source: Adapted from Fitzpatrick (2021) and García Lupato and Meloni (2023).

The electoral pillar (EL) aims to analyse the actual implementation of online voting procedures within a party. Ballots are considered digital if they are granted to all party members and take place fully online or in hybrid form (offline and online). The key indicators include: EL1 if the party has organised online voting for selecting the latest party leader; EL2 if the party has implemented online voting for all or some of the candidates selected for the last national election; EL3 if online voting for the selection of the party executive board was implemented during the last party congress.

Deliberative pillar (DEL) provides information on deliberative initiatives or tools implemented by the party: DEL1 refers to the existence of Online Participatory Platforms (OPPs) developed by the party, provided they allow sharing ideas, policy proposals or other deliberative exchanges by party members, which were in use at the moment of the coding; DEL2 is connected to one-time, specific and non-recurrent deliberative initiatives organised through an online format (e.g. a forum to discuss policy proposals before an election); DEL3 assesses if the party has carried out its most recent congress partially or totally online. This does not include just streaming, as deliberation and decision-making must also occur online.

The participatory pillar (PART) enquires about members and/or supporters’ engagement in online activities promoted by the party, including voting on consultations, as well as participation in digital activities for electoral campaigns or other internal and external party activities. PART1 considers if the party (at different levels) has fully or partially organised an online direct vote to decide on certain proposals or issues (e.g. joining the government, approving the manifesto or voting on strategic decisions); PART2 assesses if the party allows registering as a member or supporter through online supports exclusively, without any necessary face-to-face step. For example, via website or email; PART3 encompasses both activities to connect with voters or to coordinate party members. This indicator includes online canvassing, meetings or other online activities for campaigning; PART4 refers to other digital initiatives promoted inside the party (e.g. webinars, training, etc.); PART5 assesses if the party promotes digital activities, such as online petitions and protests. This indicator also enquires if a party's own activities are through non-partisan webs or social media; PART6 include non-electoral activities carried out by the party in (commercial) digital platforms.

The resources pillar (SOURCE) covers different online initiatives to gather economic resources such as SOURCE1 states if the party membership fee payment can be paid entirely through the website; SOURCE2 considers if the party allows for online crowdfunding, donations or microcredits; SOURCE3 reports if there is an official online store of the party.

The communication pillar (COM) is the most complex one and includes several indicators to communicate with voters and media: COM1 reports if the party has a website; COM2 states if the party is present in any Social media platform; COM3 assess if the party has an account on one or more Social Networking Sites (SNS) (e.g. Facebook, Instagram, X, Tik Tok). COM4 states if the party uses Mobile Instant Messaging Services (MIMS) (e.g. WhatsApp, Telegram, Signal); COM5 considers if the party uses other forms of online communications such internet channels (YouTube, Twitch), official blogs or online newsletters. COM6 assesses if the party broadcasted online its most recent congress; COM7 contacting the party, reports if the party offers a section or form through which citizens can directly send a message (e.g. through the website); COM8 reports if the party offers a specific website section or form through which citizens can directly send a message to the leader or candidate; COM9 shows if the party website displays information regarding news, agenda or future events.

The DIGIPART dataset also provides a set of calculated indicators, built upon the sum of different components. There is one indicator per each pillar (Electoral Index, Deliberative Index, Participatory Index, Resources Index and Communication Index). These are calculated by adding the different sub-pillars of each dimension. Moreover, there are two more comprehensive indicators: one the one hand, the Party Digitalisation Index (PD index) accumulates all 5 indexes into one overall index of internal digitalisation and provides a general figure of how digitalised parties are (Cronbach’s alpha= 0.83). On the other hand, the Digital Democratic Innovations Index (PDDI Index) is based on the addition of all those dimensions referring to Digital Democratic Innovations (Electoral, Deliberative and participatory indexes).

## 4 Experimental Design, Materials and Methods

The DIGIPART dataset was conceived as a pilot study to develop a protocol, codebook and dataset to comparatively analyse party digitalisation in some European countries after the COVID-19 pandemic. A codebook and an excel file with all the indicators and some specific examples on how to code them was developed at the beginning of the process. Different meetings were conducted to check for potential disagreements in interpreting and coding some indicators. Only when all questions were cleared, the coding started.

The case selection of the dataset faced two main problems: first, there was no previous comparative information (or index) available on party digitalisation in Europe, which complicated any kind of potential sampling or the development of Most Similar/Different System Designs (digitalised vs. non digitalised parties). Second, the research team pooled a limited number of resources that substantially constrained the potential ambition of the data collection. Hence, we employed the Digital Economy and Society Index (DESI), developed by the European Commission since 2014, as a (very limited) proxy for conducting the case selection. We opted for some of the larger European countries with higher DESI scores (Table 3). Such selection still included a diverse range of political systems, party systems and parties. We believe that the size of our selected countries (in terms of population, economic and political power) makes them very central to any analysis of political digitalisation in Europe. This indeed provides valuable insights into how party digitalisation has been implemented in some countries that do not face significant technological limitations. However, it is important to note that some research questions may not be fully addressed and that making inferences to smaller or less digitalised countries would be problematic (see next section).

**Table 3:** Descriptive statistics of the DIGIPART dataset (v1.1) by indexes and countries.

	N	Minimum	Maximum	Mean	Std. Deviation
EL Index	76	0	3	0,56	0,82
DEL Index	76	0	3	0,8	1
PART Index	76	0	5	2,61	1,38
SOURCE Index	76	0	3	1,25	0,81
COM Index	76	0	9	6,68	1,58
PD Index	76	0	23	17,12	7,43
PDDI Index	76	0	11	3,97	2,58

	Spain	France	UK	Germany	Italy
EL Pilar	35,29 %	0,00 %	33,33 %	40,74 %	0,04 %
DEL Pilar	23,53 %	14,29 %	33,33 %	77,78 %	33,33 %
PART Pilar	69,41 %	40,00 %	72,00 %	68,89 %	33,85 %
SOURCE Pilar	41,18 %	38,10 %	60,00 %	74,07 %	25,64 %
COM Pilar	76,47 %	72,22 %	83,33 %	93,83 %	63,68 %

In each of the selected countries (Germany, Italy, France, Spain, and the United Kingdom ) we coded information of all statewide parties with representation at the lower house, meaning parties that operate across the entire territory of a national state [24]. To expand the scope of the dataset, in the Version 1.1, we also added those non-statewide parties that, during the data-gathering process achieved representation at the national level (e.g. lower chamber). The first version of the DIGIPART dataset (v0) contained data from 62 parties. Version 1.1. of was enlarged with new data on non-statewide parties across the different countries (*n* = 76).

The timeframe of the analysis is right after the COVID-19 pandemic. By that time many political parties substantially changed and adapted their organization through digital tools to facilitate internal communication, participation and decision making [25,26]. The timeframe of the analysis is also connected to the moment when the fieldwork was conducted, between July 2021 and September 2022.

The data was collected through two main processes: on the one hand, tracking the public information offered by parties themselves, in particular their websites and manifestos. On the other hand, press repositories and daily journals were consulted to complement or contrast the previous information. This included the own parties’ press releases, as they normally inform of the different processes they develop. In a limited number of cases, when information was not available from the party, the coders used newspaper coverage to understand how the process developed, always relying on quality press. Due to the nature of the variables addressed, this technique was used as a residual measure to confirm the coding.

To ensure consistency in the coding process, several steps were implemented: first, the codebook was developed and discussed among all coders, ensuring a clear understanding of each variable. Second, most variables were conceived as numerical and directly observable. To simplify the process, all indicators were presented in a dichotomous way: 0 if the digital initiative did not exist, 1 if it existed, and a dot ( . ) if the evidence was unsure or not found. As the variables were focused on the absence or presence of an activity based on documentary evidence, there was little room for interpretation, which reduced potential coding discrepancies. Third, following the codebook, a country expert coded one or several parties from that given country. The evidence and results of such coding process were carefully revised by other coders, that eventually raised potential discrepancies. Fourth, all issues or queries were collectively discussed, and, when needed, coding guidelines were clarified to enhance consistency. Hence, the coding process of this pilot dataset is the result of a collective deliberative process instead of the outcome of several individual coding processes (See the next section to discuss issues with this methodological approach).

Nonetheless, it is important to state that the parties in the sample have been subject to changes after the coding, and our data only covers one point in time. If some of the developments have been extended or suppressed after the data collection ended, these changes are not registered.

## Limitations

The DIGIPART dataset in its current version (v. 1.1) captures a specific moment in time, compiled between July 2021 and September 2022. This introduces three potential limitations: first, the dataset is not suitable for diachronic analysis; second, attempting to gather data from previous years poses significant challenges, especially given that the data encompasses 76 parties and has been coded through documentary evidence (not opinions or experts). Data compilation may have been influenced by COVID-19, as parties may have digitalised certain procedures that were later de-digitalised once the pandemic subsided. These changes, whether extending or suppressing certain digitalisation initiatives, are not captured in the current dataset until future waves of data have been collected.

The case selection presents some limitations, as well. The dataset was not conceived through Most Similar/Different Systems Design because very little systematic information was available on party digitalisation beforehand. Hence the idea of developing a pilot study. In addition, Central and Eastern Europe countries, smaller and less digitalised countries or other regions of the world are not included, which may impact certain analysis and limits the possibility of making inferences. However, the research team is planning future waves to broaden the number of countries, variables and party information over time.

The aim of the pilot study was to capture whether (or not) digital tools were involved in certain party activities. That shaped the way the variables were conceptualised and coded. Binary (yes/no) answers indeed present some limitations for assessing the intensity of digitalisation. More nuanced ways to understand the phenomenon might be needed for future research. In addition, an extended version of the dataset should rely on different coders per country, so coder inter-reliability tests could be performed.

Finally, the dataset was built before the widespread development of Artificial Intelligence (AI). Further theorisation is needed to better understand in which dimensions and how AI is affecting political parties. That should inform future versions of DIGIPART.

## Ethics Statement

The authors have read and follow the ethical requirements for publication in Data in Brief and confirm that the current work does not involve human subjects, animal experiments, or any data collected from social media platforms relating to individual people.

## Credit Author Statement

**Marco Meloni:** Conceptualisation, Methodology, Data curation, Validation, Writing - original draft, Supervision. **Fabio G. Lupato:** Conceptualisation, Methodology, Data curation, Validation, Writing - review & editing. **Felix von Nostitz:** Conceptualisation, Methodology, Validation, Funding acquisition, Writing - review & editing. **Giulia Sandri:** Conceptualisation, Methodology, Validation, Writing - review & editing.**Oscar Barberà:** Conceptualisation, Methodology, Data curation, Validation, Funding acquisition, Writing - review & editing, Supervision. **Adrià Mompó:** Data curation, Validation, Writing - review & editing.

## Data Availability

ZenodoDIGIPART - Digitalisation in Parties dataset (Original data)
